# ICC-dementia (International Centenarian Consortium - dementia): an international consortium to determine the prevalence and incidence of dementia in centenarians across diverse ethnoracial and sociocultural groups

**DOI:** 10.1186/s12883-016-0569-4

**Published:** 2016-04-21

**Authors:** Henry Brodaty, Claudia Woolf, Stacy Andersen, Nir Barzilai, Carol Brayne, Karen Siu-Lan Cheung, Maria M. Corrada, John D. Crawford, Catriona Daly, Yasuyuki Gondo, Bo Hagberg, Nobuyoshi Hirose, Henne Holstege, Claudia Kawas, Jeffrey Kaye, Nicole A. Kochan, Bobo Hi-Po Lau, Ugo Lucca, Gabriella Marcon, Peter Martin, Leonard W. Poon, Robyn Richmond, Jean-Marie Robine, Ingmar Skoog, Melissa J. Slavin, Jan Szewieczek, Mauro Tettamanti, José Viña, Thomas Perls, Perminder S. Sachdev

**Affiliations:** Dementia Collaborative Research Centre – Assessment and Better Care, School of Psychiatry, UNSW Medicine, The University of New South Wales, Sydney, Australia; Centre for Healthy Brain Ageing (CHeBA), School of Psychiatry, UNSW Medicine, The University of New South Wales, Sydney, Australia; Psychogeriatric Mental Health and Dementia Service, St Vincent’s Hospital Sydney, Darlinghurst, Australia; New England Centenarian Study, Geriatrics Section, Department of Medicine, Boston Medical Center, Boston University School of Medicine, Boston, Massachusetts USA; Department of Medicine, Albert Einstein College of Medicine, Bronx, USA; Department of Genetics, Albert Einstein College of Medicine, Bronx, USA; Department of Public Health and Primary Care, Institute of Public Health, Cambridge University, Cambridge, UK; Sau Po Centre on Ageing, The University of Hong Kong, Hong Kong, SAR China; Department of Social Work and Social Administration, The University of Hong Kong, Hong Kong, SAR China; Department of Neurology, University of California Irvine, Irvine, USA; Department of Epidemiology, University of California Irvine, Irvine, USA; Graduate School of Human Sciences, Clinical Thanatology and Geriatric Behavioral Science, Osaka University, Suita, Japan; Gerontology Research Centre, Lund, Sweden; Centre for Supercentenarian Research, Keio University School of Medicine, Tokyo, Japan; Department of Clinical Genetics, VU University Medical Centre, Amsterdam, The Netherlands; Alzheimer Centre, Department of Neurology, VU University Medical Centre, Neuroscience Campus Amsterdam, Amsterdam, The Netherlands; Department of Neurobiology and Behavior, University of California Irvine, Irvine, USA; Department of Neurology and Biomedical Engineering, Oregon Health and Science University, Portland, USA; Neuropsychiatric Institute, Prince of Wales Hospital, Randwick, Australia; Laboratory of Geriatric Neuropsychiatry, Department of Neuroscience, IRCCS - Istituto di Ricerche Farmacologiche Mario Negri, Milan, Italy; Department of Medical and Biological Sciences, University of Udine, Udine, Italy; AAS 1 Triestina, Trieste, Italy; Department of Human Development and Family Studies, Iowa State University, Ames, USA; Institute of Gerontology, University of Georgia, Athens, Georgia USA; School of Public Health and Community Medicine, UNSW Medicine, The University of New South Wales, Sydney, Australia; National Institute on Health and Medical Research, INSERM, Paris, France; Department of Psychiatry, Sahlgrenska University Hospital, Gothenburg, Sweden; Department of Geriatrics, School of Health Sciences in Katowice, Medical University of Silesia, Katowice, Poland; Department of Physiology, University of Valencia and INCLIVA, Valencia, Spain

**Keywords:** Centenarians, Dementia, International, Prevalence, Incidence, Risk factors

## Abstract

**Background:**

Considerable variability exists in international prevalence and incidence estimates of dementia. The accuracy of estimates of dementia in the oldest-old and the controversial question of whether dementia incidence and prevalence decline at very old age will be crucial for better understanding the dynamics between survival to extreme old age and the occurrence and risk for various types of dementia and comorbidities. International Centenarian Consortium – Dementia (ICC-Dementia) seeks to harmonise centenarian and near-centenarian studies internationally to describe the cognitive and functional profiles of exceptionally old individuals, and ascertain the trajectories of decline and thereby the age-standardised prevalence and incidence of dementia in this population. The primary goal of the ICC-Dementia is to establish a large and thorough heterogeneous sample that has the power to answer epidemiological questions that small, separate studies cannot. A secondary aim is to examine cohort-specific effects and differential survivorship into very old age. We hope to lay the foundation for further investigation into risk and protective factors for dementia and healthy exceptional brain ageing in centenarians across diverse ethnoracial and sociocultural groups.

**Methods:**

Studies focusing on individuals aged ≥95 years (approximately the oldest 1 percentile for men, oldest 5th percentile for women), with a minimum sample of 80 individuals, including assessment of cognition and functional status, are invited to participate. There are currently seventeen member or potential member studies from Asia, Europe, the Americas, and Oceania. Initial attempts at harmonising key variables are in progress.

**Discussion:**

General challenges facing large, international consortia like ICC-Dementia include timely and effective communication among member studies, ethical and practical issues relating to human subject studies and data sharing, and the challenges related to data harmonisation. A specific challenge for ICC-Dementia relates to the concept and definition of’abnormal’ in this exceptional group of individuals who are rarely free of physical, sensory and/or cognitive impairments.

## Background

The prevalence of dementia in centenarian studies varies widely from 27 % (or 42 % once drop-outs were accounted for [1]) to 76 % [2] and even 85 % (albeit in a small sample [3]). Reasons for this variability include small sample sizes, non-ascertainment of all centenarians within a selected region, the healthy volunteer effect, non-inclusion of residents in skilled nursing facilities, refusal of proxy-consent by ‘protective’ family members, frequent shift in residence owing to care needs, and other potential biases. Longitudinal studies suffer the limitation of selective attrition, particularly due to high mortality in such an elderly sample. Additionally, not all studies demand adequate proof of age, a problem particularly relevant when the claimed age is greater than 110 years old ([4, 5]).

In the context of dementia diagnosis, accurate cognitive assessments of centenarians can be challenging due to decreased stamina and difficulty with hearing and/or vision, and most studies, at least to date, have not used an adequately sensitive battery of tests to rule out false negative results. Differences in cognitive assessment tools, variability in diagnostic criteria, and difficulty in selecting an appropriate comparison group are further challenges in comparing and/or merging results from different studies. Birth year cohort-specific influences are likely to also explain differences between studies, with accumulating evidence that the age-specific incidence of dementia may be decreasing in high income countries [6]. Cho et al. [7] found that a later cohort of centenarians was significantly better off in mental, physical, social and economic domains. A recent UK study found later-born individuals to have a lower risk of dementia [8]. Steen [9] found similar cohort effects in five different cohorts of 70 year olds, with later cohorts performing significantly better than their earlier counterparts, although a Swedish study [10] found no differences in cognitive function between two cohorts. Influences of environment, such as climate [11] and geography, including rural versus urban [12], may need to be taken into consideration when examining dementia prevalence and its determinants in different populations. Dietary factors may also play a role, however data remain insufficient to date.

Given the limitations of prevalence data, it can be argued that incidence data on dementia might offer a better metric to examine the cognitive profile of this age group. However, only a handful of studies have provided incidence data on dementia in the oldest-old [13], most of which have few participants at the oldest ages and sometimes all participants aged over 90 years are combined into a single age category. A review, with sufficient age-specific data, contends that dementia incidence increases in the age range of 95–115 years [13]. A North American study, with baseline ages of 90–103, also observed an increase in dementia incidence with age [14]. Other studies that include participants aged 95 or over, observed a *slowing* of the age-related increase in incidence of dementia [15, 16], and a decline in rates for men [17, 18]. Finally, a study that included the oldest 0.01 percentile (e.g. currently 110+ years old) has demonstrated the progressive compression of both disability and morbidity (in 6 diseases including dementia) with older ages of survival beyond 100 years [19]. Given these different results further examination of the incidence of dementia for males and females for different age groups among the oldest ages is warranted.

Another approach taken by investigators is to examine specific cognitive functions in this group. Although relatively unexplored in the oldest old, it appears that episodic memory continues to decline through the 10th and 11th decades of life [20] with processing speed [21] and attention [22] being particularly susceptible to ageing. By contrast, many aspects of language [23] as well as executive functions [24] may remain intact with increasing age, although the data are limited by small sample sizes. The domains of cognitive function most susceptible to advanced ageing, and their magnitude of decline, have important bearing on the differentiation of the transition from normal cognition to mild cognitive impairment and dementia. In general, the patterns appear similar to cognitive changes observed in younger cohorts [25], although much more extensive evaluation of this dynamic is needed.

Some research has been conducted on risk and protective factors of dementia onset in centenarians, but little is known about the course and rate of this decline [26]. The limited data available suggest that some exceptionally long-lived individuals share risk factors for dementia with their younger counterparts [26], such as African American race [27], low education [27], smoking [28], and poor physical health as evidenced by strength, balance and gait measures [29]. Conversely, a well-known genetic risk factor for Alzheimer’s disease, the apolipoprotein E ε-4 allele, is rare amongst centenarians [30, 31]. Similarly, a number of risk factors for cardiovascular disease, although consistent in predicting cognitive impairment in younger cohorts [32], have different effects on cognition in older populations [33]. Interestingly, a number of studies recently demonstrated that centenarians have frequencies of disease-associated genetic variants that are similar to the general population [34, 35]. Yet, as noted above, people achieving ages over 105 years tend to avoid or delay such age-related diseases [19]. Bergman and colleagues [36] noted this “buffering effect” of certain genes previously, however limited research exists on the relation of this effect to dementia.

Most existing studies of the oldest old are necessarily small, limiting the power to appropriately examine prevalence and incidence data, cognition and risk factors. Data harmonisation across numerous studies is a cost-effective approach with increased statistical power that offers the potential to explore both existing and novel research questions. Harmonising data across studies to create a single, large database permits evaluation of both study-level and individual-level effects, and the direct comparison of results across studies with opportunity for immediate evaluation of differences, when found, and additional analyses to reconcile such differences [13, 27]. It is important to note that the provenance and contextual information of each study must be taken into account in any such analysis. Other benefits of collaboration include accelerated accumulation of scientific knowledge and enhanced generalisability associated with using a larger heterogeneous sample [13].

In the proposed consortium of centenarian and near-centenarian studies, comprising at least fifteen datasets from Asia, Europe, the Americas, and Oceania, The Dementia Harmonisation Project of the International Centenarian Consortium (ICC-Dementia https://cheba.unsw.edu.au/group/icc-dementia) plans to address the epidemiological challenges confronting the study of this exceptional group of individuals. The Consortium’s objectives are to: (i) determine the sex specific and percentile of survival-standardised prevalence and incidence of dementia and likely dementia type at the extreme tail of survival; (ii) delineate subgroups of cognitive function and their specific patterns of cognitive decline; (iii) identify associated risk and protective factors for dementia and healthy brain ageing that cross or do not cross ethnic and cultural lines; and (iv) examine the influence of contextual factors such as population ageing, survival rates and differential causes of death in different countries on the cognitive health of the oldest old.

## Methods

### Membership

Studies are eligible to participate in ICC-Dementia if they meet the following criteria: (i) the focus is on individuals aged ≥95 years; (ii) have a minimum sample of 80 individuals; (iii) assessment includes measures of cognitive function; and (iv) informed consent allows for de-identified data sharing with academic partners. Official enrolment in ICC-Dementia involves a lead investigator’s signed memorandum of understating (MOU) to share de-identified raw and/or processed data for mega-analyses as well as comparative analyses. All participating studies have the opportunity to participate in the analyses and to propose specific projects and papers. ICC-Dementia was established in 2012, as part of the long established International Consortium of Centenarian Studies (ICC), and has stated interest in participating from the 17 studies listed in Table 1. At the time of writing this report, data had been received from eight of these studies and others were in the process of submitting institutional review board protocols.

**Table 1:** Contributing Centenarian Studies

Study	Setting	Sample of centenarians	Age range	Females (%)	Race/Ethnicity	Start/end date	Reference	Centenarians per 100,000 people (year of estimate)
Sydney Centenarian Study (SCS)^a^	Sydney, Australia	345+	95–106	72	Caucasian	2008-ongoing	[48]	18.75 (2010) [55]
New England Centenarian Study (NECS)	Boston, USA	1500+	100–119	76	Caucasian	1994-ongoing	[56, 57]	22.6 (2014) [58]
Georgia Centenarian Study (GCS)	Georgia, USA	381	98–110	82	Caucasian African American	1988–2009	[59]	22.6 (2014) [58]
Tokyo Centenarian Study (TCS)^a^	Tokyo, Japan	304	100–108	79	Japanese	2000–2002	[20, 45, 60]	42.76 (2013) [61]
Swedish Centenarian Study (SwCS)^a^	Southern Sweden	100	100–101	82	Caucasian	1987–1992	[39]	20.1 (2014/15) [62]
Gothenburg 95+ Study (Go95+)^a^	Gothenburg, Sweden	1020	95–109	82	Caucasian	1996–2015	[63]	20.1 (2014/15) [62]
90+ Study (90+)^a^	California, USA	960	95–107	79	Caucasian	2003–2007	[64]	22.6 (2014) [58]
Cognitive Function and Aging Study (CFAS)	England and Wales, UK	700	95–100+	NA	Caucasian	1991–1994	[65]	21.49 (2013) [66]
Longevity Gene Project (LGP)^a^	New York, USA	462+	95–110	75	Caucasian	1998-ongoing	[67]	22.6 (2014) [58]
Five Country Oldest Old Project (5COOP)	Montpellier, France	1241	100	80	Danish (251), French (211), Japanese (337), Swedish (274), Swiss (168)	2011–2014	Personal correspondence	NA
Polish Centenarian Study (PCS)^a^	Katowice, Poland	86+	99–105	81	Caucasian	2007-ongoing	[68, 69]	8.015 (2010) [70]
Spanish Centenarian Study (SpCS)	Valencia, Spain	47+	98–107	74	Caucasian	2010-ongoing	[71]	26.44 (2013) [72]
Oregon Brain Aging Study (OBAS)	Oregon, USA	156+	55–111	60	299 Caucasian, 2 Native American, 3 Asian	1989-ongoing	[73]	22.6 (2014) [58]
Monzino 80+ Study (M80+)	Varese Province, Italy	542+	95–107	71	Caucasian	2002-ongoing	[74]	29.42 (2014) [75]
Hong Kong Centenarian Study (HKCS)	Hong Kong	153	95–108	78	Chinese	2009–2011	[76–78]	26.73 (2011) [79]
Centenarians at Trieste (CaT)	Trieste, Italy	100+	100+	90	Caucasian	2014-ongoing	[80]	29.42 (2014) [75]
The 100+ Study (100+)	Amsterdam, The Netherlands	133+	100–115	75	Caucasian	2013-ongoing	Personal correspondence	12.4 (2014) [81]

^a^data received

### Structure of the consortium

Each study is invited to provide one member for the Steering Committee, which leads the scientific agenda of the consortium and provides governance. Rules of participation approved by the Steering Committee include regulations about approval of projects, timelines for analyses, presentations and publications. New member studies are invited by the Steering Committee by consensus. Periodic teleconferences are planned to discuss scientific and administrative issues of the consortium, with some special teleconferences and/or videoconferences to deliberate case vignettes and agree upon common criteria. Consortium members plan to meet face-to-face once a year at the annual meeting of the International Centenarian Consortium, or another suitable international meeting, and most recently met in June 2015 in Sardinia, Italy.

### Website

A website has been constructed and is an information resource for potential members and the general public, https://cheba.unsw.edu.au/group/icc-dementia. A secure section of the website will be used for data queries.

### Ethics

The ICC-Dementia project has been approved by the Human Research Ethics Committee of The University of New South Wales, Sydney. Member studies are responsible for obtaining approval, when considered necessary, from their local institutional review board for the sharing of data.

### Data harmonisation

ICC-Dementia faces numerous challenges associated with data harmonisation, many of which have been previously described [27, 37]. The major challenge stems from differences between studies in design, language used for assessment, the measurement instruments used and/or differences in how questions from similar instruments are worded and responses are categorised. Cultural factors influence many of the measures and the response bias of participants. Attempts to maximise the number of studies contributing to a final dataset can require that complex information be simplified, e.g., converted from a continuous measure to a categorical scale. Although there is a possible reduction in validity involved in simplifying data, there are mechanisms by which this can be tested (see below) [27].

ICC-Dementia will harmonise demographic data, scores on screening measures of cognitive function (e.g. Mini-mental State Examination, MMSE [38]), neuropsychological test data and measures of functional status. Data received will be handled according to the following protocol:

#### Demographic data

All studies provide age in years, birth year and sex; these variables will require minimal recoding to common scales. Education is categorised into a four-level scale of the highest level of education achieved (less than high school completion, high school completion, technical or college diploma, university degree).

#### Measures of cognitive status

Table 2 shows the cognitive and functional measures available from each of the participating studies. Score ranges and means will be reported for all measures, with appropriate cut-offs derived using all available data from each contributing study. Where there is no overlap in specific measures between studies, when possible, published data on equivalence of scores and thresholds for different measures are used. For example, the MMSE was not administered in the Swedish Centenarian Study [39], so equivalence of scores on the Berger Scale [40, 41] are used.

**Table 2:** Mental status and dementia scales used by contributing studies

Study	SCS	NECS	GCS	TCS	SwCS	Go95+	90+	CFAS	LGP	5COOP	PCS	SpCS	OBAS	M80+	HKCS	CaT	100+
Mental status or cognitive scales																	
MMSE	✓	✓^a^	✓	✓		✓	✓	✓	✓	✓	✓	✓	✓	✓	✓	✓	✓
ACE-R	✓																
CPRS						✓											
CAMCOG								✓									
CASI-S							✓										
ADL scale	✓	✓	✓	✓	✓	✓	✓	✓		✓	✓	✓	✓	✓	✓	✓	✓
Neuropsychological data	✓	✓^a^	✓	✓^a^	✓	✓	✓	✓		✓			✓	✓		✓	✓
Dementia scales																	
CDR	✓	✓^a^	✓	✓		✓	✓			✓			✓			✓	
GDS			✓	✓								✓	✓				
BDS		✓												✓			
Berger					✓												
MDRS		✓^a^				✓											
AGECAT								✓									
CAMDEX								✓									

*ACE-R* Addenbrooke’s Cognitive Examination, *AGECAT* Automated Geriatric Examination for Computer Assisted Taxonomy, *BDS* Blessed Dementia Scale, *CAMDEX* Cambridge Examination for Mental Disorders in the Elderly, *CAMCOG* the cognitive and self-contained part of the Cambridge Examination for Mental Disorders of the Elderly, *CASI-S* Cognitive Abilities Screening Instrument-Short, *CDR* Clinical Dementia Rating Scale, *CPRS* Cognitive Participation Rating Scale, *GDS* Global Deterioration Score, *MDRS* Mattis Dementia Rating Scale, *MMSE* Mini-Mental State Examination

^a^Data only available for a subset of the sample

#### Harmonisation plan for neuropsychological test performance

Different studies’ test batteries differ in the tests included, versions of the same test, and methods of administration and/or scoring. Inspection of the range of tests available from each study led to the decision to obtain scores from as many studies as possible for the following five domains: memory, attention/processing speed, language, executive function and visuospatial/constructional function. Tests are allocated to domains consistent with common practice [42, 43]. To harmonise neuropsychological test scores across studies, raw scores are converted to Z-scores, adjusted for age, sex and education, using the means and standard deviations (SD) of an appropriate reference group. Most contributing studies that have neuropsychological data use a contemporaneous younger, culturally-equivalent cohort as the reference group to norm centenarian data; we will obtain these data from each contributing study. For studies that may not have these data available, we source a younger, culturally equivalent cohort as a reference group. This can be achieved using data from another consortium within our group, i.e. the Cohort Studies of Memory in an International Consortium (COSMIC) [44], where data have already been received from 11 different countries. Performance on a test or in a cognitive domain is regarded as impaired or exceptional if its Z-score, calculated using the mean and SDs of the reference group, is less or greater than 1.5 SD from the mean.

#### Functional data

Contributing studies differ in their assessment of functional ability both in instruments used and in areas of functional ability assessed, e.g. basic versus instrumental activities of daily living. In order to harmonise data, the most common and compatible items are chosen to form harmonised variables [45], although this requires judgement by an assessor which may be subjective.

#### Harmonisation of risk factor variables

We harmonise risk variables using standard definitions for hypertension, diabetes, high cholesterol, alcohol use, body mass index (BMI), etc. Some of the procedures, developed as part of our multiple longitudinal studies (Sydney Memory and Ageing Study (MAS) [46], Older Australian Twins Study [47] and Sydney Centenarian Study [48]) have been used previously in a project where MAS data were harmonised with data from the Australian Imaging Biomarker and Lifestyle (AIBL) Study [40].

#### Dementia diagnoses

Dementia, or Major Neurocognitive Disorder as designated in DSM-5, is diagnosed on the basis of DSM-IV [49] and DSM-5 [50] criteria within a subsample of the consortium studies. Diagnoses are made via an algorithmic approach supported by clinical consensus meetings for five of the consortium studies. Participants are categorized as having dementia if they exhibit significant cognitive impairment from a previously stable level and these cognitive deficits are interfering with independence in everyday living. Cases are brought to consensus meetings if the diagnoses differ from those given previously by the study investigators, or if other factors, e.g. sensory or motor problems impacting performance, are present and not easily incorporated into an algorithmic approach. Expert panels comprising physicians (minimum of two neurologists, neuropsychiatrists, psychogeriatricians or geriatricians) and one or more neuropsychologists make consensus diagnoses, using all available clinical, neuropsychological, laboratory and imaging data to do so. The panel takes into account cross-cultural issues, fatigue, multi-morbidity and sensory abnormalities. Inter-rater reliability between panels is established with a representative set of 20 cases being reviewed by all panels.

### Statistical analyses

#### Prevalence and incidence data

Overall rates from the different studies will be age-standardised to allow direct comparisons to be made between studies with different age distributions and sampling methods. The age distribution of the sample formed by pooling all participating studies will be used as the standard distribution to which all studies will be adjusted. Participants will be divided into 5-year age categories and rates will be obtained for each of these age ranges. The standardized rate (SR) for each of the studies will be calculated using the following formula: SR = (SUM (ri x Pi))/SUM Pi, where ri is the rate in each of the 5-year age ranges, Pi is the proportion of the population in the standard distribution in each of the 5-year age ranges, and SUM is the sum of values over each of the age ranges, “i”. Birth years of the various cohorts will be taken into account.

#### Cognitive data

For the analysis of patterns of change in cognition over time, as measured by continuous variables with distributions close to that of the normal distribution, Linear Mixed Modelling (LMM) will be employed. This procedure will minimise bias due to non-random attrition, and allow whole-of-sample analyses. For the analysis of non-normally distributed continuous variables, these can be transformed to approximate the normal distribution better using the Box-Cox procedure so that LMM procedures can be used. Alternatively, Generalized Linear Mixed Modelling (GLMM) can be used with an appropriate link function (e.g. loglinear, negative binomial) depending on the nature of the distribution. To allow for asynchronous measurement occasions across studies, time will be modelled as a continuous independent variable, with non-linear effects examined by the introduction of polynomial power terms into the equation. The compression of morbidity hypothesis will be investigated by examining the correlation between the age of onset of dementia (determined both historically and by examining incident cases) with the time interval between the onset and the time of death. A negative correlation would provide support for the hypothesis.

#### Risk factors

For the examination of risk factors for dementia, survival analysis using Cox regression, and also GLMM, will be used. For the GLMM analyses, the logit link function will be used to accommodate the binary outcome variable. Both procedures allow for whole of sample analyses to minimise bias due to selective attrition over time, and reduce the loss of power due to smaller samples if only cases with complete data were used. For both procedures, risk factors will be entered as independent variables, together with other appropriate control variables (sex, age and education). To examine whether the operation of risk factors vary across studies or different national-cultural groups, such factor(s) will be included in the equations, together with interactions between these factors and the risk-factor variables.

## Discussion

### Challenges

General challenges facing large international consortia have been described previously [51]. These include funding, timely and effective communication among member studies in different countries and, data harmonisation. Specific challenges for ICC-Dementia relate to the identification of cognitive and functional impairment and the diagnosis of dementia in this exceptional group of long-lived individuals where testing is subject to numerous constraints and normative data are lacking. Additionally, the included studies vary in their age ranges, with most including both nonagenarians and centenarians. Considering that nonagenarians (5th-15th percentile of survival for men and women born in 1900 and thereafter) are increasingly common and centenarians (<1 percentile of survival) are still relatively rare, they likely represent different phenotypes in terms of underlying environmental and genetic influences and therefore risks of age-related diseases and disability [52]. Thus, grouping nonagenarians with centenarians, or claiming that a study of primarily nonagenarians is a study of the oldest 1 percentile runs the risk of missing important differences. We recently reviewed the many challenges of diagnosing dementia in this group and proposed some solutions [53]. Although the approaches for diagnosing dementia in centenarians vary widely, the essential denotation of a dementia diagnosis is that the individual has experienced a decline in cognitive function from a previous level to the extent that their independence in everyday activities has been affected and this decline is not better explained by some other physical or mental disorder. Challenges for ICC-Dementia include operationalising cognitive decline, defining what is ‘normative’ [53], attributing functional impairment to cognitive decline in the presence of physical and sensory impairments [54], and doing this for populations that differ greatly in education, language and cultural expectations.

### Future projects

Future ICC-Dementia projects are planned which endeavour to make comparisons between cohorts, countries and ethnic groups. These include: (i) the cognitive profile and trajectory of cognitive decline in centenarians and near centenarians; (ii) risk and protective factors for dementia and exceptional healthy brain ageing; and (iii) where possible, biomarkers (e.g. blood, genetic and MRI-derived) of dementia in the oldest old.

It is expected that future work will investigate more refined and specific topics addressing the overall objectives of ICC-Dementia. These could include systematic investigation of the ‘compression of morbidity hypothesis’ and the association between cognitive and physical frailty. These projects will be enabled and facilitated by increasing the number of ICC-Dementia member studies to ensure that there are sufficient data on variables not collected by all studies. The ICC-Dementia is open to membership to other studies that meet the eligibility criteria. We envision the ICC-Dementia setting the stage for an ambitious well-coordinated study of cognitive impairment and dementia in the oldest old.

## Conclusions

The accuracy of estimates of the number of people with dementia and the unanswered question of whether dementia continues to rise with age are crucial for health planning and care of the very old. The identification of cohort-specific and non-specific influences on survival to very late life and risk factors for dementia will provide insights into underlying mechanisms of delaying or escaping common dementias. The dementia-free centenarians, and those who delay the onset of dementia until very late in life, can serve as models of healthy brain ageing, potentially providing insights for the entire population.

## Declarations

### Ethics approval

ICC Dementia was approved by the Human Research Ethics Committee at the University of New South Wales. https://research.unsw.edu.au/human-research-ethics-home.

### Consent for publication

Not applicable.

### Availability of data and materials

Not applicable.

## Abbreviations

ADL: activities of daily living
AIBL Study: Australian Imaging Biomarker and Lifestyle Study
APOE: apolipoprotein E
BMI: body mass index
COSMIC: Cohort Studies of Memory in International Consortium
GLMM: Generalized Linear Mixed Modelling
ICC-Dementia: International Centenarian Consortium–Dementia
LMM: Linear Mixed Modelling
MAS: Sydney Memory and Ageing Study
MMSE: Mini-mental State Examination
SD: standard deviation
